# Discovery and characterization of miRNA genes in atlantic salmon (*Salmo salar*) by use of a deep sequencing approach

**DOI:** 10.1186/1471-2164-14-482

**Published:** 2013-07-17

**Authors:** Rune Andreassen, Merete Molton Worren, Bjørn Høyheim

**Affiliations:** 1 Faculty of Health Sciences, Oslo and Akershus University College of Applied Sciences, Oslo, Norway; 2 Bioinformatics Core Facility, Ifi, University of Oslo, Oslo, Norway; 3 Norwegian School of Veterinary Science, BasAM-Genetics, PO Box 8146 DEP, Oslo NO-0033, Norway

## Abstract

**Background:**

MicroRNAs (miRNAs) are an abundant class of endogenous small RNA molecules that downregulate gene expression at the posttranscriptional level. They play important roles in multiple biological processes by regulating genes that control developmental timing, growth, stem cell division and apoptosis by binding to the mRNA of target genes. Despite the position Atlantic salmon (*Salmo salar*) has as an economically important domesticated animal, there has been little research on miRNAs in this species. Knowledge about miRNAs and their target genes may be used to control health and to improve performance of economically important traits. However, before their biological function can be unravelled they must be identified and annotated. The aims of this study were to identify and characterize miRNA genes in Atlantic salmon by deep sequencing analysis of small RNA libraries from nine different tissues.

**Results:**

A total of 180 distinct mature miRNAs belonging to 106 families of evolutionary conserved miRNAs, and 13 distinct novel mature miRNAs were discovered and characterized. The mature miRNAs corresponded to 521 putative precursor sequences located at unique genome locations. About 40% of these precursors were part of gene clusters, and the majority of the *Salmo salar* gene clusters discovered were conserved across species. Comparison of expression levels in samples from different tissues applying DESeq indicated that there were tissue specific expression differences in three conserved and one novel miRNA. Ssa-miR 736 was detected in heart tissue only, while two other clustered miRNAs (ssa-miR 212 and132) seems to be at a higher expression level in brain tissue. These observations correlate well with their expected functions as regulators of signal pathways in cardiac and neuronal cells, respectively. Ssa-miR 8163 is one of the novel miRNAs discovered and its function remains unknown. However, differential expression analysis using DESeq suggests that this miRNA is enriched in liver tissue and the precursor was mapped to intron 7 of the transferrin gene.

**Conclusions:**

The identification and annotation of evolutionary conserved and novel *Salmo salar* miRNAs as well as the characterization of miRNA gene clusters provide biological knowledge that will greatly facilitate further functional studies on miRNAs in this species.

## Background

MicroRNAs (miRNAs) are an abundant class of endogenous small RNA molecules. They regulate gene expression as part of the miRNA-induced silencing complex (miRISC) at the post-transcriptional level by binding to the mRNA of target genes in a sequence specific manner. The binding of the miRISC to mRNA results in downregulation of gene expression either by inhibition of translation or by degradation of the target genes [1,2]. Most mature miRNAs are 20–24 nt in length while precursor-miRNAs are usually 60–80 nt and have a hairpin secondary structure [3]. Some miRNAs are highly conserved from species to species while other miRNAs seems to be species specific [4,5]. They play important roles in multiple biological processes by regulating genes that control developmental timing, growth, stem cell division and apoptosis [6-9]. They are often expressed in a tissue-specific manner, and a large proportion, probably more than 30%, of all protein coding genes of animals may be regulated by miRNAs [10,11]. Failure in miRNA expression or mutation in miRNA genes may result in genetic disease. There are e.g. 163 diseases reported in the miR2Disease database that are associated with dysfunction of miRNA genes or miRNA/target gene-interaction [12]. Dysfunctional miRNA/target gene interaction may also contribute to development of cancer when miRNAs e.g. act as oncogenes [13]. On the other hand, naturally occurring variation in miRNA genes or miRNA target sites may contribute to normal phenotypic differences. Some of these phenotypic variants, like the one affecting muscularity in sheep, may affect economically important traits [14].

Recent advances in sequencing technology have led to increased sensitivity in sequencing analysis (deep sequencing) that allows even lowly abundant small RNAs to be detected [15]. Experimental data from such deep sequencing analysis together with bioinformatic tools that utilize the current knowledge about the characteristic structure of miRNA precursor molecules and how they are processed into mature miRNAs in the cell may be used in miRNA discovery projects. Combining the sensitive deep sequencing methods and these tools it is possible to discover both novel and evolutionary conserved miRNAs [16-18].

Despite the position Atlantic salmon (*Salmo salar*) has as an economically important domesticated animal, and despite the focus on functional genomics in aquaculture, there has been little research on miRNAs in *Salmo salar*, and in miRBase (http://www.mirbase.org/), there are at present no *Salmo salar* miRNAs. The regulatory role of miRNAs in growth, in the immune system or in other developmental and physiological processes in salmon is therefore unknown. However, the fact that heptamers identical to known miRNA binding sites are conserved in the 3′ UTRs of *Salmo salar* genes [19], and that homology based *in silico* studies indicate that there are many miRNA genes in the salmon genome [20] both suggest, as expected, that miRNAs are important regulators that control a large proportion of protein coding genes also in Atlantic salmon.

Due to a relatively recent whole-genome duplication (WGD) believed to have occurred between 25 and 120 million years ago in the common salmonid ancestor the salmon genome is complex [21]. Present salmonids appear to have retained more than 50% of loci as duplicates, also referred to as ohnologs i.e. duplicate genes that originate from a WGD [22,23]. Many of the miRNAs that are evolutionary conserved across species would therefore be expected to exist as duplicate gene copies (ohnologs) in *Salmo salar*. Ohnologs may be deleted or develop into pseudogenes, but they also have the potential to gain new function. It has been suggested that WGD could allow for a more rapid evolution of novel miRNA families, although evolutionary studies of ancient vertebrate WGDs have not supported such a hypothesis [24,25]. Since Atlantic salmon is a vertebrate species with an additional and recent WGD, studies of miRNAs in this species might contribute to our biological knowledge on the fate and evolution of miRNAs following whole genome duplications.

Furthermore, knowledge of miRNAs and their target genes may in the future be used to control health and to improve performance of economically important traits in farmed animals and aquaculture species. Thus, the aims of this study were to identify and characterize both evolutionary conserved as well as putative novel miRNA genes in Atlantic salmon by deep sequencing analysis of small RNA libraries. Nine different tissues were analyzed independently to identify a large number of miRNAs with a high confidence. This also allowed for a comparison between tissues to detect any obvious differences in tissue specific expression of particular miRNAs. All miRNAs discovered were mapped to genomic locations in the present version of the *Salmo salar* genome assembly. The subsequent comparison of miRNA precursor locations allowed us to map miRNA clusters in the salmon genome.

## Results and discussion

### Discovery and characterization of miRNAs in *Salmo salar*

Total RNA from liver (two samples), spleen (two samples), kidney, head kidney, heart, brain, intestine, white muscle and gills from individuals at the pre-smolt developmental stage as well as total RNA from a one day old individual was successfully extracted. The concentration of total RNA ranged from 58–900 ng/μl (total volume 100 μl). Following size separation and preparation of the libraries the twelve tissue and developmental stage specific libraries were successfully subjected to next generation sequencing using Illumina Genome Analyzer IIx sequencing platform. The number of quality filtered and adaptor trimmed reads from each sample ranged from 1.4 to 18 millions while the number of unique reads ranged from 64593 to 246444. An overview of total read numbers in the twelve samples together with concentrations of total RNA in the extracts is given in Table 1.

**Table 1:** **Summary of samples sequenced for discovery of*salmo salar*miRNA genes**

**Tissue, gender**	**Sample Id**	**Conc ng/μl**	**#Total trimmed reads**^**1**^	**#Total size filtered reads**^**2**^	**#Unique reads**^**3**^	**Acc# NCBI SRA**^**4**^
Liver ♀	1	772	1,446,902	1,101958	64593	SRR866573
Liver ♂	6	404	1,647,133	1,175579	75273	SRR866579
Spleen ♂	2	114	8,597,057	7,027915	295940	SRR866583
Spleen ♀	4	58	2,236,013	1,856468	89165	SRR866587
Kidney ♂	3	428	10,065,660	8,180104	243430	SRR866589
Head kidney ♀	7	521	7,375,957	4,687901	246444	SRR866590
Heart ♀	8	252	2,812,993	2,058117	118366	SRR866605
Brain ♂	10	324	6,331,448	4,770906	132558	SRR866611
Intestine ♂	11	946	12,428,822	9,849737	197094	SRR866612
White muscle ♂	12	495	5,972,384	2,788579	142444	SRR866613
Gills ♂	22	340	6,240,735	5,058051	132038	SRR866614
One day old individual	13	475	18,041,561	12,824661	172048	SRR866615

^1^Total number of quality filtered and adaptor trimmed reads in a sample.

^2^Total number of quality filtered and adaptor trimmed reads in size interval 17-30 nt in a sample.

^3^Number of unique reads in a sample.

^4^Accession number to sequencing data from each of the samples in NCBI SRA database.

All miRNAs discovered were initially identified by miRDeep analysis [26]. The underlying idea of the miRDeep software package is to detect miRNAs by comparing reads from deep-sequencing data to how miRNA precursors are processed in the cell. The final output from miRDeep analysis was a list of putative miRNA precursors with their corresponding 5p and 3p mature reads that was assigned a miRDeep score (score estimated as described in [16]). All outputs with precursors that showed a score value above the lower threshold (see Methods) and with reads that were perfect matches to the expected 5p and 3p mature miRNAs processed from that particular precursor were considered as putative miRNA genes.

**Figure 1:**
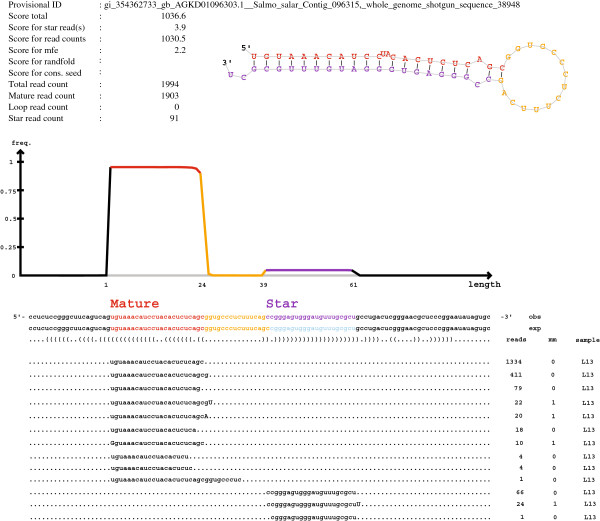
**The figure illustrates the output from a miRDeep2 analysis.** The lower part of the figure shows a 113 nt sequence (putative miRNA precursor) from *Salmo salar* contig 096315 of the preliminary genome assembly along with reads that aligned to this sequence. The experimental data (reads) were from sample 1 (liver). The total miRDeep2 score for this miRNA was 1036 (based on scores for hairpin stability (mfe) and the read counts). Total number of different unique reads that aligned to the putative precursor is listed in first column on the right side of the figure. The sequence of the mature 5p miR is shown in red while the sequence of the mature 3p miR is shown in purple. A graphic illustration of the hairpin-loop from this particular precursor is shown on top of the figure. The subsequent homology analysis against miRBase confirmed this precursor as a true miRNA gene belonging to the 30 family of evolutionary conserved miRNA genes.

Figure 1 shows an example of a putative *Salmo salar* miRNA precursor sequence as reported by the initial miRDeep analysis. The reads are shown as “stacks” below the precursor sequence and, as demonstrated in the figure, they align to the precursor in a discrete manner and close to the 5′ end (5p reads) or 3′ end (3p reads) of the putative precursor sequence. The number of each of the unique 5p and 3p reads that align to the precursor is given in the figure, and in this case the read numbers suggested that the 5p reads, being approximately 20× more frequent, were the predominant mature miRNAs while the 3p reads were the less frequent mature miRNAs processed from this precursor. Most reads (both 5p and 3p) align to the precursor from identical 5′ nucleotides. The experimental data in this case supported that this was a true miRNA precursor with the corresponding processed mature miRNAs.

All putative precursor sequences discovered by the miRDeep analysis were further analyzed by BLAST homology searches against the stem-loop sequences in miRBase (see Methods). Any precursor that provided a match with an expectation value less that 1× e^-7^ against a stem-loop sequence in miRBase was considered a true *Salmo salar* ortholog of an evolutionary conserved miRNA gene. Any *Salmo salar* miRNA belonging to miRNA families conserved across species were therefore first identified in an initial miRDeep analysis that was independent of cross-species comparisons. Then the homology based identification approach (BLAST analysis against miRBase) provided further support that they were true miRNAs belonging to a certain evolutionary conserved miRNA family.

A total of 180 distinct evolutionary conserved mature 5p-miRNA sequences and their corresponding 3p-miRNA sequences were identified in this two-step approach together with 356 different putative precursor sequences (mirs) at unique positions within the preliminary assembly of the *Salmo salar* genome. In addition, a total of 111 of these 356 precursors had one identical copy at another unique genome location, while fifteen precursors had three identical copies and one precursor had four identical copies at unique genome locations. There were thus a total of 501 putative precursors discovered at unique genome locations that corresponded to the 180 conserved mature miRNAs. An overview of all such evolutionary conserved precursor (mir) sequences identified along with their corresponding mature 5p and 3p sequences is given in Additional file 1.

Any precursor that provided a significant match to a stem-loop sequence of a certain miRNA gene family in miRBase was assumed to be a *Salmo salar* ortholog of this miRNA gene. Consequently, they have been annotated as a *Salmo salar* precursor belonging to this miRNA family in Additional file 1. Many of the mature sequences within the same family of miRNA genes revealed small sequence difference. These were annotated using the same family number but differed by adding lettered suffixes (−a, -b etc.) in accordance with the nomenclature rules (see Methods and [4,27]).

The annotation of miRNAs showed that we have identified 106 evolutionary conserved miRNA families in our material. The type of evolutionary conserved miRNA genes present in a species is expected to be in accordance with its taxonomic level. All 106 miRNA families identified are among those expected to be present in teleosts while those miRNAs suggested to be specific to Mammalia, Tetrapoda and Amniota [25] were not observed.

The result from the miRDeep analysis showed that there were several additional putative precursor sequences revealing scores above threshold and with reads that aligned as expected for true 5p and 3p mature miRNAs. However, homology analysis showed that they did not belong to any of the miRNA gene families in miRBase. These putative novel miRNAs were further analyzed by BLAST searches against the preliminary salmon genome sequence assembly. Those putative precursor sequences that provided multiple significant hits against the salmon genome sequence (more than 5 hits with E-values <1× e^-6^) were removed (data not shown). These sequences had initially been identified as putative precursors since reads aligned discretely and they had the ability to form stable stem loops. However, being present in multiple copies in the salmon genome they were considered likely to be some kind of interspersed repeats and/or long tandem repeats. The reads that matched these sequences did, however, align in a discrete manner and, thus, have the properties expected for processed small RNAs. We can, therefore, not rule out that they may represent some kind of functional small RNAs in *Salmo salar*[28,29].

The remaining putative precursors were analyzed against other small RNA databases and the refseq-RNA database in Genbank (see Methods), but did not match other kinds of noncoding small RNAs or mRNAs. They did, however, exhibit the following common properties. The precursors along with the reads were detected in at least two samples, and, in all cases, reads that perfectly matched the expected mature miRNAs (5p and 3p) were detected. Finally, most of the reads showed the properties expected from products of processed precursors, aligning from identical nucleotide positions at their 5′end. They meet the consensus criteria used to recognise novel miRNAs [30], and are likely to be true novel miRNAs. A total of 13 distinct novel mature 5p-miRNAs and their corresponding 3p-miRNAs and 15 different putative precursors, some being present as identical duplicates, were discovered in our material. Table 2 gives an overview of all the putative novel miRNA precursors and their 5p and 3p mature miRNAs. The novel miRNAs with their precursor and mature sequences as well as their genome locations is also given in Additional file 2. All miRNAs discovered have been submitted to miRBase. The data from this study has also been submitted to the NCBI SRA database (accession # SRP022967). The accession numbers to data from the individual samples are given in Table 1.

**Table 2:** Novel non-conserved miRNAs discovered in this study

**miRNA**	**Mature-5p**	**Mature-3p**	**Precursor (hairpin, mir)**	**No. loci**	**Tissues**
ssa-mir-7132b	gacuuggucaaagcuccucagc	ugaggcguuuagaacaaguuca	5′-gacuuggucaaagcuccucagcagauguuucagagaaucc**ugaggcguuuagaacaaguuca**	4	multiple
ssa-mir-8156	guccugacuguccugacuguc	cugucaugaccguccugacugu	5′-**guccugacuguccugacuguc**uuuaccguacuggcugucaugaccguccugacugu	1	multiple
ssa-mir-8157	uagcacauuacaguacagcugu	acucugugcucugcugugcugu	5′-uagcacauuacaguacagcuguauuguuacugcacugu**acucugugcucugcugugcugu**	2	multiple
ssa-mir-8158	aagagguuucacacauacaaau	uuuguacgugugaaacuucuucc	5′-aagagguuucacacauacaaauguuaauuuuaaaauacuca**uuuguacgugugaaacuucuucc**	1	multiple
ssa-mir-8159	ucaguaacuggaaucugucccugc	agggccggcugguuacugcgc	5′-**ucaguaacuggaaucugucccugc**agacacacuguagagcagggccggcugguuacugcgc	1	multiple
ssa-mir-2184	aacaguaagaguuaaugugcug	gcacguaggcucuuacaguaca	5′-**aacaguaagaguuaaugugcug**gguucucaaucagcacguaggcucuuacaguaca	1	multiple
ssa-mir-8160	agaauaaugccagcagucggcc	ccagcacugguguuauuggga	5′-**agaauaaugccagcagucggcc**cugguuuccuaggggccagcacugguguuauuggga	1	multiple
ssa-mir-8161	agaauaaugccagcagucggcc	ccagcgcugguguuauuggga	5′-**agaauaaugccagcagucggcc**auauugucucaaaggccagcgcugguguuauuggga	2	multiple
ssa-mir-7552b	cuacaauuaaaggauauuucuu	aaauaucuuguaauuguuuggu	5′-**cuacaauuaaaggauauuucuu**gugacugagcauauacggaaauaucuuguaauuguuuggu	2	multiple
ssa-mir-7552a-1	uuacaauuaaaggauauuucuu	aaaugucccuuaauuguuuggc	5′-**uuacaauuaaaggauauuucuu**gcgaaugaaugagagacggaaaugucccuuaauuguuuggc	1	multiple
ssa-mir-7552a-2	uuacaauuaaaggauauuucuu	aaauuucccuuaauuguuuggc	5′-**uuacaauuaaaggauauuucuu**gcgauugaauaugagacggaaauuucccuuaauuguuuggc	1	multiple
ssa-mir-8162	uugucucagaccuguuugugcug	ucacaacggaucugggaucagu	5′-uugucucagaccuguuugugcuguaguuguuaucaggaug**ucacaacggaucugggaucagu**	1	multiple
ssa-mir-8163	uuucugaccaugugaccuggggg	ucaggucacauguucaggaua	5′-uuucugaccaugugaccugggggccgagaguguuuccacc**ucaggucacauguucaggaua**	1	liver
ssa-mir-8164	cagagguauuguaauaucguga	acgauacggcgauaauucugau	5′-cagagguauuguaauaucgugauacuacaauacucacc**acgauacggcgauaauucugau**	1	multiple

^1^The short processed miRNA with largest number of read counts is indicated by bold letters within precursor sequences.

Taken together we have identified 180 distinct evolutionary conserved miRNAs and 13 distinct novel miRNAs. The precursor sequences of the evolutionary conserved mature miRNAs were distributed in multiple genomic locations and corresponded to a total of 501 putative precursors discovered at unique genome locations. Only 44 out of the 180 distinct conserved mature sequences (25%) corresponded to one single precursor located at one unique genome location while the others (75%) corresponded to precursors located in more than one genome location (either two identical precursors or slightly different ones matching identical mature miRNAs). Thus, about three quarters of the distinct conserved mature sequences could be transcribed from multicopy miRNA genes. Many of these precursors may, however, be transcriptionally inactive pseudogenes. The corresponding percentage of such multicopy miRNA genes in zebrafish (*Danio rerio*) was reported by Chen et al. [29] to be about 44% (68 out of 153). This could indicate that a larger percentage of evolutionary conserved miRNA genes exist as multicopy genes in *Salmo salar*. A larger copy number of those miRNA genes that are conserved across species would be in agreement with previous studies indicating that about 50% of the *Salmo salar* genome consists of duplicate sequence originating from the salmonid specific genome duplication [21].

However, to assemble the salmon genome sequence with its large amount of highly similar duplicate sequences is challenging, and the preliminary genome assembly may be of a relatively poor quality. Thus, we cannot rule out that some of the precursors now assigned to different contigs i.e. appears as duplicated at different unique genome locations, may in fact not be true duplicates, but just single locus sequence that has been incorrectly assigned into different contigs. The distinct mature novel miRNAs, on the other hand revealed a somewhat different precursor distribution. Eight out of the thirteen distinct mature miRNAs (61%) corresponded to one single precursor found in one unique genome location. One would expect miRNAs that have evolved after the salmonid specific genome duplication to be present in a lower copy number than the evolutionary conserved ones. This finding is, thus, in agreement with the expectations for these miRNAs and supports the fact that they could be true novel salmon specific miRNAs.

There is usually an arm selection when precursors are processed leading to a high copy number of products from one arm and much less frequent number of mature products from the other arm. While most miRNAs show arm selection it has been reported that in some miRNA genes there may be a less pronounced difference in expression of the mature products with similar copy numbers of the 5p and 3p mature miRNAs (see e.g. [17]). To assess any dominance in arm preference among the mature miRNAs in our material we compared read counts of mature miRNAs aligning to either 5p or 3p for all evolutionary conserved miRNAs discovered. We found only a few cases with similar copy numbers of mature miRNAs from both arms while there was a 5p arm dominance in approximately 60% of all cases and 3p arm dominance in the remaining cases. A similar distribution of either 5p or 3p arm dominance was observed in the group of distinct novel miRNAs. Also in this group there were slightly more cases where mature miRNAs were processed from the 5p arm (55% of the cases). These distributions correspond well with observations from other studies, e.g. Li et al. [17], and in Additional files 1 and 2 the arm dominance (5p or 3p) is given for each miRNA.

### miRNA gene clusters discovered in *Salmo salar* are evolutionary conserved

Clusters of miRNA genes have been reported in several species including medaka (*Oryzias latipedes*) and zebrafish (*Danio rerio*) [17,29]. A miRNA gene cluster is, according to miRBase, defined as two or more miRNA genes located less than 10 kb apart and with same direction of the transcription. Applying this definition a total of 198 of the precursors discovered (approximately 40% of the precursors) were located in a gene cluster, and there were a total of 84 gene clusters. One of the novel miRNA genes discovered (ssa-mir 2184) was located in a gene cluster with two other conserved miRNA genes (ssa-mir 212a-2 and 132–2). The remaining miRNA genes that were clustered were from the group of evolutionary conserved miRNAs. Most of the gene clusters consisted of two precursors, but from three to six clustered precursors was also observed. Together, a total of 87 distinct mature miRNAs may be transcribed from clustered precursors. The distances between precursors in our clusters were in most cases small and most often less than five kb. This is half of the size distance usually used for defining gene clusters. However, a miRNA gene cluster can only be detected in the salmon genome when located within a contig, and the total size of most contigs of the preliminary genome assembly is rather small (the contig N50 is 9.3 kb). This limits the ability to discover gene clusters in the *Salmo salar* genome. The number of gene clusters may therefore be underestimated in our material. A complete overview of all gene clusters, their locations within a contig and the Genbank references to these contigs is given in Additional file 3.

Apart from eight gene clusters that were discovered in one contig only, the clustered miRNA genes could be subdivided into 20 groups where the members of each group consisted of the same miRNA gene families clustered in the same direction but observed in different contigs. There were from two to nine contigs showing such similar miRNA gene-clusters within each of the 20 groups in our material. These groups are indicated by roman numerals in Additional file 3.

Multiple copies of certain miRNA gene clusters within a genome have also been observed in other species [17,29]. However, a comparison with results from *Danio rerio* (see Table 3) showed that, in general, there was a larger duplicate number in *Salmo salar*. Again, considering the unfinished state of the preliminary salmon genome sequence, it is possible that some of these duplicate gene clusters are not true duplicates, but located in single sequence loci that have been incorrectly assigned into different contigs.

**Table 3:** **Gene clusters in*Salmo salar*that are conserved across species**

**Ssa mir gene clusters**	**Copy # S. salar**^**1**^	**Dre-mir gene clusters**	**Copy # D. rerio**^**2**^	**Chrom. locations D. rerio**^**3**^
Ssa-mir: 17–3, 18b, 19a-1, 20a-3, 19c-3, 92a-2	7 contigs	Dre-mir: 17a-1, 18a, 19a, 20a, 19b, 92a-1	2	1, 9
Ssa-mir: 18a, 20b	2 contigs	Dre-mir: 18c, 20b, 19c, 363	1	14
Ssa-mir: 462a, 731	1 contig	Dre-mir: 462, 731	1	8
Ssa-mir-222a-2, 221	1 contig	Dre-mir: 222a, 221	1	9
Ssa-mir: 106a-1, 93a-1, 25-1	6 contigs	Dre-mir: 93, 19d, 25	1	14
Ssa-mir: 194a-2, 192a-2, 192b	2 contigs	Dre-mir: 194a, 192	1	10
Ssa-mir: 183–3, 96–1, 182	4 contigs	Dre-mir: 183, 96, 182	1	4
Ssa-mir: 144, 451a-1, 451a-1	2 contigs	Dre-mir: 144, 451	1	5
Ssa-mir: 212a-1, 132-1	4 contigs	Dre-mir: 212, 132-1	1	15
Ssa-mir: 23a-4, 27a-1, 24b	9 contigs	Dre-mir: 23a-1, 27e, 24-4	6	22, 10, 3, 3, 2, 8
Ssa-mir: 430a, 430c, 430b	2 contigs	Dre-mir^4^: 430a-18, 430c-1, 430b-4 …	1	4
Ssa-mir: 130d-2, 301c	6 contigs	Dre-mir: 130c-1, 301c	2	10, 10
Ssa-mir: 99–1, let-7c-1	4 contigs	Dre-mir: 99–1, let-7c-1, 125c	2	15, 10,
Ssa-let: 7 g-1, 7a-5	4 contigs	Dre-let: 7e, 7a-5	2	23, 4
Ssa-let: 7a-1, let-7f	1 contig	Dre-let: 7a-1, 7f	1	11
Ssa-let: 7e-2, 7 h	2 contigs	Dre-let: 7 g-2, 7 h	2	23
Ssa-mir: 30d-1, 30a-4	5 contigs	Dre-mir: 30d, 30b	2	16, 13
Ssa-mir: 100a-1, 7-a-3	4 contigs	Dre-mir: 100–1, let-7a-2, 125b-1	2	15, 5
Ssa-mir: 15e, 16-a-2	4 contigs	Dre-mir: 15a-1, 16b	3	1, 15, 9
Ssa-mir: 199a-1, 214-2	2 contigs	Dre-mir: 199–1, 214	1	20
Ssa-mir: 29a, 29b-2	2 contigs	Dre-mir: 29b-2, 29a	1	4
Ssa-mir: 200b-1, 429	2 contigs	Dre-mir: 200b, 200a, 429a	1	23
Ssa-mir: 1–2, 133-4	2 contigs	Dre-mir: 1–2, 133a-1	1	Zv9_scaffold_3540
Ssa-mir: 143, 145-2	1 contig	Dre-mir: 143–1, 145	1	14
Ssa-mir: 181b, 181a-5	1 contig	Dre-mir: 181a-2, 181b-2	1	8
Ssa-mir: 193, 365-2	2 contigs	Dre-mir: 193a-1, 365-2	2	3, 6

^1^Number of different contigs in S. salar with similar precursors clustered in same transcriptional direction.

^2^Number of clusters in D. rerio with similar precursors in same transcriptional direction.

^3^Chromosomal location of miRNA gene clusters in D. rerio.

^4^Multiple 430 family genes in one large cluster.

To reveal whether the particular gene clusters discovered in Atlantic salmon were present in other vertebrate species we compared the 28 different *Salmo salar* miRNA gene clusters to the ones discovered in *Danio rerio* (miRBase and [29]) and humans (data from miRBase). These comparisons showed that 26 out of the 28 *Salmo salar* miRNA gene clusters discovered were also observed in other vertebrates. Table 3 shows the 26 different gene clusters from Atlantic salmon together with the orthologous gene clusters in *Danio rerio*. These comparisons showed that most of the gene clusters discovered in Atlantic salmon are conserved across vertebrate species. The fact that these particular miRNA precursors discovered in our material are located in clusters as reported in other vertebrates support that they are true *Salmo salar* miRNA genes.

### Tissue specific expression differences and functional predictions

The Illumina® TruSeq Small RNA sample preparation protocol is designed to provide data that may be used to profile expression levels of miRNAs (see Methods). To test the performance of the method directly in our material we performed a linear regression analysis of normalized read count levels (see Methods) of all miRNAs in the two liver samples. This analysis showed a Pearson correlation coefficient (r) of 0.97 indicating that the method applied reproduced the different expression levels of the individual miRNAs very well. Although expression differences could be measured more accurately and confirmed by additional quantitative analysis, we believe that large differences between individual miRNAs from different tissue samples in our present material would suggest that they are expressed in a tissue specific manner. A few such differences were observed when applying DESeq (see Methods) to compare miRNAs expressed in one tissue to all other tissues. One such miRNA, ssa-miR 736, revealed a normalised read count of 261 in the heart tissue sample (sample 8, Table 1). No ssa-miR 736 reads were detected in any of the other tissue samples. Applying the DESeq package (see Methods) to further evaluate the observed difference in ssa-miR 763 expression showed that the difference was significant (P-adj = 0.02). Studies in other vertebrates have shown that miR 736, a gene conserved across species, belongs to the 208 family of miRNA genes. This family of miRNA genes is specifically expressed in cardiac tissue. It is therefore often referred to as “myomiR” [31]. Our observation suggests that ssa-miR 736 has a similar tissue specific function in *Salmo salar*.

One may assume that the miRNA gene clusters that are observed across species (same miRNA genes and transcriptional directions) are evolutionary conserved as clusters because they are important key genes in regulatory gene networks that are essential to all vertebrates. The evolutionary conserved *Salmo salar* miRNA gene clusters could therefore be expected to have similar regulatory functions in this species as in other vertebrates. One could, from such an assumption, predict that the ssa-mir-15e, 16a-2 gene cluster regulate cell cycle progression [32] while the genes in the ssa-mir-144, 451a-1, 451a-1 gene cluster is likely to regulate erythropoiesis [33]. Such predictions would be more robust if there were additional experimental evidence that supported the assumed function of a given *Salmo salar* gene cluster. The miRNA 212 and 132 gene cluster is known to be important in neurological development and time perception, and due to these important functions their mature miRs are enriched in neuronal cells [34-36]. Interestingly, the largest number of reads that perfectly matched the mature reads from the clustered ssa-miR genes 212 and 132 was observed in brain tissue (sample 10, Table 1). Applying the DESeq package the normalized read counts were 13860 and 5293 for miR 212 and 132, respectively. In contrast, normalized read counts in the other tissue samples were 72 and 44 for miR 212 and 132, respectively (log2 fold changes of approx. -7). The following comparison of brain tissue to the other tissues showed that the differences for both miRNAs were significant (P-adj = 0.003). The results suggest that the salmon miRNAs belonging to 212 and 132 families are more extensively expressed in brain tissue. Thus, in the case of these genes, both the fact that they are clustered and the fact that they showed an elevated expression in brain tissue indicate that they have similar developmental and regulatory functions in *Salmo salar* neuronal cells as revealed in other vertebrates [34-36].

One of the novel miRNAs discovered, ssa-miR 8163, was observed in liver tissue (sample 1 and 6, Table 1), and at low level in the sample from the one day old individual (consisting of a mix of all tissues, sample 13, Table 1) while no ssa-miR 8163 reads were detected at all in any of the other samples. Applying DESeq the normalized read counts in liver (two samples combined) was 241 while they were absent in samples from other tissues. Further analysis showed that the difference was significant (P-adj = 0.049). This suggests that there may be a higher expression level of this novel miRNA in liver tissue. The precursor sequence of this miRNA (ssa-mir 8163) was present as a single copy sequence in the salmon genome assembly. To retrieve more information about the genome location of this miRNA gene we performed a BLAST analysis against the nt/nr-database in Genbank. This homology analysis revealed an almost perfect match (97% identity) to intron number 7 in the transferrin gene of *Oncorhynchus tshawytscha* (Genbank: AH008271, basepairs 2808–2868, E = 2× e^-19^). Thus, the gene is located in, and presumably co-transcribed with, the transferrin gene that is known to be under positive selection among salmonids [37].

## Conclusion

This study provides the mature miRNAs and their precursor sequences to a large number of conserved *Salmo salar* miRNAs. Thirteen distinct novel mature miRNAs were also discovered. The comparison of precursor locations within the salmon genome revealed a large number of evolutionary conserved *Salmo salar* miRNA gene clusters. Together, these results provide knowledge on miRNAs in Atlantic salmon that will greatly facilitate further functional studies in this economically important species.

## Methods

### Small RNA isolation and deep sequencing

Two pre-smolt Atlantic salmon (*Salmo salar*) individuals were dissected and tissue from the different organs was collected for isolation of total RNA. Liver and spleen were collected from both individuals (one sample from each) while one sample from one individual was collected from kidney, head-kidney, heart, brain, gills, white muscle and intestine. A one day old individual was also homogenized and used for isolation of total RNA. Thus, a total of twelve samples from nine different tissues were included in our material. Total RNA was isolated from each of the samples by use of the mirVana miRNA isolation kit (Ambion) following the manufacturer’s protocol. The RNA concentration and purity were determined using the Bioanalyzer and the RNA 6000 Nano Assay (Agilent) following the manufacturer’s protocol. The Illumina® TruSeq Small RNA sample preparation protocol was used to prepare the libraries (http://nextgen.mgh.harvard.edu/attachments/TruSeq_SmallRNA_SamplePrep_Guide_15004197_A.pdf). The protocol takes advantage of the natural structure common to most known microRNA molecules. Most mature miRNAs have a 5′-phosphate and a 3′-hydroxyl group as a result of the cellular pathway used to create them. Because of this, the Illumina adapters are directly, and specifically, ligated to miRNAs. After RT-PCR using the specific adapters supplied with the kit the samples were size separated in a 6% polyacrylamid gel to enrich for small RNA molecules prior to sequencing. The library construction and size separation was performed at the Norwegian Genomics Consortium’s genomics core facility followed by sequencing analysis using Illumina’s Genome Analyzer II × sequencing platform.

### Pre-processing and analysis of sequencing data

The 32 bp sequence reads (fastq-files) from each of the 12 samples were quality filtered and adaptors (5′ TGGAATTCTCGGGTGCCAAGG 3′) were removed by use of the fastx-clipper tool (http://hannonlab.cshl.edu/fastx_toolkit/). Any adaptor sequences of two or more nucleotides were removed. Consequently, if a read started or ended in one base identical to the adaptor it was not removed.

The miRNA discovery analysis were independently performed in each of the 12 samples to allow for detection of miRNAs expressed only in particular tissues. Version 0.1 of the *Salmo salar* genome sequence from ICSASG (International Cooperation to Sequence the Atlantic Salmon Genome), GenBank accession number: GCA000233375.1, was used as a reference genome. The high quality, adaptor processed reads were used as the experimental data, and analysis was performed against the salmon reference genome using the miRDeep2 software package (mapper and miRDeep2 analysis modules) [16,26]. Default commands were used in the miRDeep2 analysis except that conservation scoring was omitted and the parameter g was set to −1 to allow all precursors to be analyzed during automatic excision gearing.

In short, the mapper module align the reads to the genome sequence and if a larger number of reads were aligned in a discrete manner (stacks) the surrounding genome sequence (approximately 100 bp) was further analyzed (miRDeep2 module) with regard to miRNA precursor gene characteristics (e.g. ability to fold into a hairpin). The statistics of the read positions and frequency of reads within a stable hairpin are scored and combined into a total miRDeep score that is a measurement of the posterior probability that the hairpin is the stem-loop of a true miRNA gene. A signal-to-noise ratio that estimate total miRNA hairpins reported/mean estimated false positive miRNA hairpins over 100 rounds of permutated controls is assigned to the varying miRDeep2 log-odds score cut-offs. We used the miRDeep2 score that yielded a signal-to-noise ratio of 40:1 as a cut-off threshold. All precursors with scores above the threshold were, thus, regarded as putative miRNAs. These were further analyzed by BLAST searches using the putative precursor sequences as input against all known stem-loop sequences from all species deposited in miRBase, release 19 (http://www.mirbase.org/search.shtml). We defined a significant hit as any precursor that provided a match with an E-value ≤ 1× e^-7^ to a stem-loop in the database. A putative miRNA from miRDeep2 analysis that provided a significant hit in the BLAST analyses against miRBase was accepted as a true *Salmo salar* miRNA and annotated as the *Salmo salar* ortholog of the miRNA gene in miRBase that retrieved the best hit.

Any miRNAs with identical mature sequences that mapped to two (or more) slightly different precursor sequences were considered to be iso-miRNA genes. They were assigned the same family number, but differed by numbered suffixes (e.g. ssa-mir-145-1 and ssa-mir-145-2). Any miRNAs with precursors that matched the same families, but with a small difference in nucleotide sequence of the mature sequences were considered to belong to the same family and differed by lettered suffixes (e.g. ssa-mir-15a and ssa-mir-15b). Some precursors revealed at different loci were entirely identical when comparing their sequences. These were considered to be duplicates of identical miRNA genes. The nomenclature rules used were in agreement with guidelines from miRBase [4,27].

The precursors identified by mirDeep that did not provide a significant match to miRBase were considered as putative novel miRNAs. All such precursors were used as queries in BLAST analysis that were performed against the nt/nr and refseqRNA databases in Genbank (http://blast.ncbi.nlm.nih.gov/), the functional small RNA database ver. 3.4 (http://www.ncrna.org/frnadb/blast), and the collection of small RNA families in Rfam (http://rfam.sanger.ac.uk/). Any putative precursor or mature sequences that provided significant hits against these databases were considered to be other kinds of small RNA and excluded. Finally, all precursors were used as queries in BLAST analysis against the *Salmo salar* genome sequence, assembly ver.01 (http://www.arkgenomics.org/tools/blastpage?species=salmon). Any putative precursor that provided significant BLAST hits, defined as E-value ≤ 1× e^-6^ against multiple loci (>5) in the salmon genome reference sequence were considered to be part of interspersed repeats or tandem repeats and, consequently, excluded as putative novel miRNAs. The remaining putative novel miRNAs were validated in the following manner: they should be detected in at least two independent experiments, the reads should match perfectly expected mature products from both arms (5p and 3p) and the reads should support that there were a consistent processing of the 5′-end of the mature sequences. Passing all these criteria they were considered to be novel miRNAs.

A simple analysis of the presence or absence of reads that were identical matches to the mature miRNA consensus sequences of the miRNAs discovered was performed for each sample. This exercise was performed to reveal whether there were any miRNAs that showed evidence of being expressed in only one or a few particular tissues.

Four miRNAs (miRNAs 736, 212a, 132–1 and 8163) that appeared as differently expressed in particular tissues were further analyzed by use of the DESeq software package (http://www.bioconductor.org/packages/devel/bioc/vignettes/DESeq/inst/doc/DESeq.pdf) [38]. Total miRNA counts (all reads perfectly matching the miRNAs discovered in a sample) were used for normalization in DESeq. The expression of each of the four miRNAs in one particular tissue was compared to all other tissues. A P-value adjusted for multiple testing with the Benjamin-Hochberg procedure (P-adj) was used to evaluate whether there were any significant differences in the expression of the four miRNAs. A prerequisite for using the data for expression comparisons is that the method applied reproduces the different individual miRNA expression levels in a sample well. To directly measure our sequencing methods’ ability to reproduce expression levels we performed a linear regression analysis using normalized read counts from all miRNAs discovered in the two liver samples as input. The regression analysis showed a Pearson correlation coefficient (r) of 0.97 indicating that the method used reproduced the expression level (normalized read counts) of different individual miRNAs very well.

The presence of clustered miRNA genes in our material were investigated by comparing precursor locations within contigs. Any two miRNA precursors located within same contig, less than 10 kb apart and with same direction of the transcription was considered a miRNA gene cluster. This definition is the same as the one used in miRBase where 10 kb is the default value for cluster search (http://www.mirbase.org/search.shtml).

## Competing interests

The authors declare that there are no competing interests.

## Authors’ contributions

RA conceived and coordinated the study, extracted total RNA, participated in bioinformatic analyses, carried out manual processing of the data, annotation of miRNAs and submissions to miRBase and GeneBank and drafted the manuscript. MMW performed major part of the bioinformatic analyses and helped in preparation of the final manuscript. BH participated in the coordination of the study, helped in preparation of the draft manuscript and in the final manuscript. All authors have read and approved the final manuscript.

## Supplementary Material

Additional file 1 **All evolutionary conserved *Samo salar *miRNAs discovered.** Click here for file

Additional file 2 All novel miRNAs discovered. Click here for file

Additional file 3 miRNA gene clusters identified in Samo salar. Click here for file
